# Effects of Environment, Genetics and Data Analysis Pitfalls in an Esophageal Cancer Genome-Wide Association Study

**DOI:** 10.1371/journal.pone.0000958

**Published:** 2007-09-26

**Authors:** Alexander Statnikov, Chun Li, Constantin F. Aliferis

**Affiliations:** 1 Discovery Systems Laboratory, Department of Biomedical Informatics, Vanderbilt University, Nashville, Tennessee, United States of America; 2 Department of Biostatistics, Vanderbilt University, Nashville, Tennessee, United States of America; 3 Center for Human Genetics Research, Vanderbilt University, Nashville, Tennessee, United States of America; 4 Department of Cancer Biology, Vanderbilt University, Nashville, Tennessee, United States of America; University of East Piedmont, Italy

## Abstract

**Background:**

The development of new high-throughput genotyping technologies has allowed fast evaluation of single nucleotide polymorphisms (SNPs) on a genome-wide scale. Several recent genome-wide association studies employing these technologies suggest that panels of SNPs can be a useful tool for predicting cancer susceptibility and discovery of potentially important new disease loci.

**Methodology/Principal Findings:**

In the present paper we undertake a careful examination of the relative significance of genetics, environmental factors, and biases of the data analysis protocol that was used in a previously published genome-wide association study. That prior study reported a nearly perfect discrimination of esophageal cancer patients and healthy controls on the basis of only genetic information. On the other hand, our results strongly suggest that SNPs in this dataset are not statistically linked to the phenotype, while several environmental factors and especially family history of esophageal cancer (a proxy to both environmental and genetic factors) have only a modest association with the disease.

**Conclusions/Significance:**

The main component of the previously claimed strong discriminatory signal is due to several data analysis pitfalls that in combination led to the strongly optimistic results. Such pitfalls are preventable and should be avoided in future studies since they create misleading conclusions and generate many false leads for subsequent research.

## Introduction

One of the promising methods for analysis of the human genome and identification of genes and genomic regions contributing to phenotypes is the use of single nucleotide polymorphisms (SNPs). SNPs make up more than 90% of all human genetic variation and have been extensively studied for functional relationships between genotype and phenotype. The advent of high-throughput genotyping technologies has allowed fast evaluation of SNPs on a genome-wide scale at a relatively low cost [1]–[3].

During the last two years several groups reported success in using SNP genotyping assays in association studies of cancer [1], [4]–[8]. In particular, the study by Hu et al. reported a nearly perfect classification of esophageal cancer cases and controls on the basis of only SNP data from a case-control genome-wide association study [8]. Taken at face value, this result suggests that esophageal cancer is a solely genetic disease. This is contradictory to other literature in the field that emphasizes importance of environment for cancer susceptibility [9], [10]. In order to shed light on this issue, we re-analyzed the data of [8].

We identified two data analysis pitfalls in [8] that caused over-optimistic conclusions in the original paper: First, the SNP selection method was severely biased toward claiming significance for SNPs that are not truly associated with the disease. Second, both SNP selection and building of classifier model were performed on the same subjects as used for estimation of classification accuracy. Since neither cross-validation nor independent sample validation were performed, the resulting classification performance estimate was overoptimistic.

We conducted a re-analysis of the SNP and environmental data that corrects the above problems and found that the SNPs in this dataset are not statistically linked to esophageal cancer, while several environmental factors, especially family history of esophageal cancer (that potentially accounts for many environmental and genetic factors), have a modest association with the disease. We quantified the contribution of each of the factors to cancer classification and provided unbiased classification performance estimates using established unbiased data analysis protocols. Given the insignificant contribution of SNPs to cancer classification, our findings suggest that the SNPs identified in [8] lack statistical evidence for being involved in esophageal cancer.

## Materials and Methods

In all data analyses in addition to replicating the methods of [8], we used unbiased alternatives so that the effects of bias (if any) in the analysis of [8] could be quantified. The justification of unbiasedness of alternative methods is provided in the pertinent subsections below.

### Study Datasets

The data used in the present study is the same as used in the original paper [8]. The data consisted of 50 esophageal squamous cell carcinoma patients and 50 controls. The patients were diagnosed with esophageal cancer between 1998 and 2000 in Shanxi Cancer Hospital in Taiyuan, People's Republic of China. Twenty-five patients and nine controls had a positive family history of the disease. The controls were matched by age, sex, and place of residence.

The genotyping of venous blood samples for all subjects in the study was performed at the National Cancer Institute (Bethesda, Maryland) as summarized below: The germ line DNA was extracted and purified. DNA samples were subsequently prepared and assayed according to Affymetrix GeneChip Mapping Assay protocol. The 10K SNP arrays with 11,555 SNPs distributed throughout human genome were scanned and genotype calls were assigned automatically by the Affymetrix GeneChip DNA Analysis software. Four genotype calls were defined in the data: AA, AB, BB, or “no call”. More details on biological specimen collection and processing, target preparation, scanning, and genotype generation are provided in [8].

For each subject, the following five variables were also recorded: age at interview (years), tobacco use (yes/no), alcohol consumption (yes/no), family history of esophageal cancer (yes/no), and consumption of pickled vegetables (yes/no).

### SNP Array Data Preparation

Before data analyses, we preprocessed the SNP array data following the approach described in the original paper [8]. First, out of 11,542 SNPs in the original dataset, 105 SNPs were removed because they could not be mapped to human genome with NCBI build 36. Second, to minimize possible genotyping errors, 946 SNPs were removed because they were homozygous in either cases or controls. Third, for the same reason, 482 SNPs were removed because they did not satisfy Hardy-Weinberg equilibrium in the control group at the α = 0.01 level [11]. Fourth, “recessive A” encoding of SNPs (AA = 1, AB = 0, BB = 0) was implemented. After these steps, the dataset consisted of 10,009 SNPs.

Since some of the data analysis methods (e.g., Principal Component Analysis or Support Vector Machines described below) require no missing data, we imputed missing genotypes in the SNP dataset and used it whenever these methods were employed. Specifically, we used the multivariate nonparametric nearest neighbor imputation technique of [12], [13].

### SNP Selection

First, we employed the SNP selection method described in [8]: For each SNP, a generalized linear model (GLM) of the probability of cancer was fit using as predictor variables the SNP and two other variables: family history of esophageal cancer and alcohol consumption. The GLM was fit for all 100 subjects without leaving out an independent testing sample. Then a p-value was obtained based on the difference between the deviance *D_0_* of the null model without any predictor variables and the deviance *D_1_* of the fitted model. The difference *D_0_–D_1_* follows a chi-squared distribution with 3 degrees of freedom. Since the above procedure is applied to each SNP in the dataset, it is necessary to adjust for multiple comparisons to ensure that the desired proportion of false positives (0.05) is preserved. To this end, Bonferroni adjustment was performed to the significance level 0.05 of the test (i.e., instead of using the significance level 0.05, the level 0.05/number of SNPs was used instead). We refer to the above method as “GLM1”. Finally, we note that Bonferroni adjustment often provides a conservative assessment of the statistical significance and assumes that all SNPs are independent, while there exist methods that are less conservative and can be applicable when the SNPs are dependent, e.g. [14]–[16].

Since the p-value of GLM1 reflects the combined effect of the three predictor variables, it tends to be small even if the SNP does not have any effect on esophageal cancer at all. To address this problem of the original analysis, we also applied the following unbiased SNP selection method: we proceed similarly as in GLM1 except that the p-value is based on the difference between the deviance *D*'*_0_* of the model including family history of esophageal cancer and alcohol consumption and the deviance *D_1_*. The resultant statistic *D*'*_0_–D_1_* follows a chi-squared distribution with one degree of freedom, and it reflects the effect of the SNP that is being analyzed. We refer to this method as “GLM2” and show that it is indeed unbiased in the Results and Discussion section and in the Supporting Information File S1.

Finally, when fitting support vector machines (see next section) to the data, we also applied the Recursive Feature Elimination (RFE) technique that is among the best performing variable selection methods for microarray gene expression data and other high-throughput molecular datasets [17]. In brief, this method involves iteratively fitting support vector machine cancer classification models by discarding the SNPs with the smallest impact on classification and selecting the SNPs that participate in the best performing classification model. Unlike the above GLM-based methods, we applied RFE only to the training set of patients and controls during cross-validation.

### Cancer Classification Models

First, we used the classification procedure described in [8]. That is, principal component analysis (PCA) was performed on the selected SNPs, and then the first principal component was extracted and used to predict cancer status.

As a state-of-the-art alternative to the PCA-based classification procedure, we applied support vector machine (SVM) classifiers [18]. The underlying idea of SVM classifiers is to calculate a maximal margin hyperplane separating the cases and controls. To achieve non-linear separation, the data are implicitly mapped to a higher dimensional space by means of a kernel function, where a separating hyperplane is found. Subjects are classified according to the side of the hyperplane they belong to. These classification methods are commonly used for analysis of high-throughput molecular data [4], [19]–[21] and have many attractive theoretical and empirical properties. For example, they often outperform other classification methods to a remarkable degree; they are also fairly insensitive to the large variable-to-sample ratio; and they can learn very complex classification functions [18], [22]. We used the libSVM implementation of the linear SVM classifiers (www.csie.ntu.edu.tw/∼cjlin/libsvm/). We also experimented with the nonlinear SVM classifiers but they resulted in more complex models with similar classification performance.

To assess the combined performance of SNPs and environmental factors (and/or family history), we used ensemble classification methods based on SVM classifiers. We present in this paper only results for the best ensembling technique that averages predictions of the two SVM classifiers for each subject: one based on SNP data and another one based on environmental factors (and/or family history). The description and results for the other ensembling techniques are provided in the Supporting Information File S2.

### Evaluation of Classification Performance

Unlike the original study [8] that used proportion of correct classifications as the performance metric, we employed area under the ROC curve (AUC) that has more power to detect predictive signal of SNPs [23]–[25]. The ROC curve is the plot of sensitivity versus 1-specificity for a range of classification threshold values. AUC ranges from 0 to 1, with an AUC equal to 0 indicating the worst possible classifier, 0.5 representing a random (i.e., uninformative) classifier, and 1 representing perfect classification. An excellent introduction to ROC analysis for classification is provided in [25].

In order to obtain unbiased AUC estimates, the cancer classification models were built and evaluated by repeated 10-fold cross-validation procedure [26]. The repeated 10-fold cross-validation estimator of classification performance can be obtained by running regular 10-fold cross-validation procedure 100 times with different splits of data into training and testing sets and reporting the average estimate over all 100 runs. This estimator is asymptotically unbiased because the testing samples are never used to train the classifier. Furthermore, the repeated 10-fold cross-validation has much smaller variance than regular cross-validation that may be affected by a non-representative split of the data [26].

## Results and Discussion

While the prior work reported 37 significant SNPs by applying method GLM1 to the esophageal cancer SNP array dataset [8], our execution of the published protocol in [8] leads to 226 significant SNPs. The difference from the reported number of 37 SNPs is due to additional filtering step that was performed to the set of SNPs significant at the Bonferroni adjusted 0.05 α-level that was not reported in the original publication (Dr. Maxwell Lee, personal communication). Since, as we show below, an unbiased method for SNP effect assessment (e.g., GLM2) yields zero significant SNPs, any additional filtering step is superfluous, therefore we do not consider such filtering in the present work.

**Figure 1:**
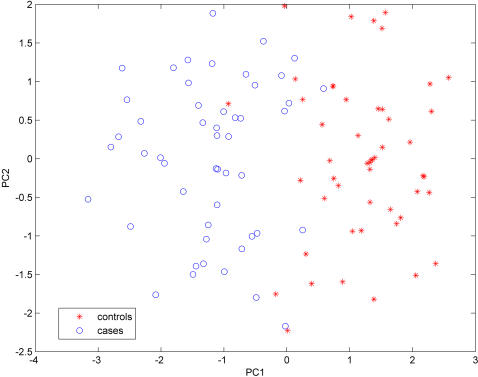
First two principal components extracted from SNPs that were selected by the method GLM1. The first principal component provides a nearly perfect separation of cases from controls.

Nevertheless, the application of the PCA-based classifier to the data of 226 significant SNPs reproduces the classification performance of the original study [8]. Namely, the first principal component provides a nearly perfect classification of patients and controls with 0.98 AUC and 0.93 proportion of correct classifications (Figure 1). However, this result is over-optimistic primarily due to the following reasons.

**Figure 2:**
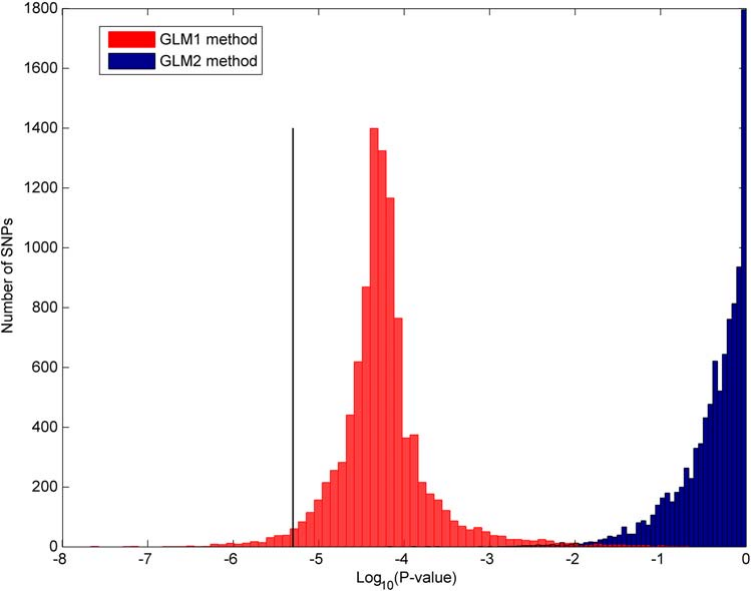
Distribution of p-values computed by GLM1 and GLM2 SNP selection methods. The figure is shown in logarithmic scale for convenience. The vertical line is the Bonferroni adjusted α-level (0.05/10,009). While there are SNPs that are significant according to GLM1 method, no SNP is significant by GLM2. The distribution of p-values for GLM2 is uniform, however the distribution for GLM1 is not.

First, the calculation of p-value in SNP selection method GLM1 does not reflect the significance of the SNP under consideration, but the significance of three variables combined (SNP, family history of esophageal cancer, and alcohol consumption). Because family history and alcohol consumption are strong risk factors for esophageal cancer, this p-value will be biased towards zero, even when the SNP has nothing to do with esophageal cancer. This bias can be demonstrated as follows: It is reasonable to assume the majority of the SNPs do not have any effect on esophageal cancer risk. For these SNPs, the p-values should follow a uniform distribution between 0 and 1. However, a vast majority of their p-values were <10^−3^ (Figure 2), which is consistent with the fact that their p-value reflected the combined effect of family history of esophageal cancer, alcohol consumption, and the SNP instead of the SNP itself. On the other hand, the procedure GLM2 reflects the effects of only SNPs and does not suffer from the above shortcoming (Figure 2). A more elaborate empirical permutation-based demonstration of why GLM1 is biased while GLM2 is not is provided in the Supporting Information File S1. The application of procedure GLM2 resulted in no significant SNPs after Bonferroni adjustment (Figure 2). Therefore, the SNPs reported in [8] as statistically significant are not statistically significant at the Bonferroni adjusted 0.05 α-level.

Second, both SNP selection by GLM1 and building of PCA-based classifier model were performed in [8] on the same 100 subjects as used for estimation of final classification accuracy. Since neither cross-validation nor independent sample validation were performed, the resulting classification performance estimate is overoptimistic as explained in [27], [28]. In order to obtain an unbiased performance estimate for the SNP selection method and classifier of [8], the above methods were applied by repeated 10-fold cross-validation. The resulting classification performance estimate was 0.68 AUC, while the original procedure in [8] led to 0.98 AUC, indicating a 0.30 AUC over-estimation.

To assess the contribution of SNPs and other variables to esophageal cancer classification, we performed several analyses that are summarized in Table 1. We used the SNP selection technique RFE [17] and the SVM classifiers [18] described in the Materials and Methods section. When SNP data is used alone, the performance is 0.51 AUC which is statistically indistinguishable from the performance of an uninformative classifier (0.50 AUC). On the other hand, four environmental variables alone (age at interview, tobacco use, alcohol consumption, and consumption of pickled vegetables) can classify cancer with 0.60 AUC indicating a modest association with cancer. When these four environmental variables are combined with SNP data, the resulting performance slightly increases to 0.62 AUC. An even more surprising result was that a single variable (i.e., family history of esophageal cancer) can classify the disease with 0.66 AUC which is more accurate than using SNP data and the four other environmental variables. We hypothesize that this happens because the family history contains information about other environmental and genetic variables that were not measured in the study data. Clearly, there are much more than four environmental variables that affect esophageal cancer. Likewise the Affymetrix 10k SNP array is an early genotyping technology that does not provide as dense genomic coverage as more recent arrays with >500k SNPs [29], [30]. When the family history is combined with other four environmental variables, cancer can be classified with 0.73 AUC which is more accurate than using either set of variables alone. On the other hand, when the family history is combined with SNP data, the resulting classifier with 0.64 AUC is not as accurate as using the former variable alone. Finally, when SNPs and all other variables are combined, cancer can be classified with 0.73 AUC.

**Table 1:** Estimates of classification performance obtained by repeated 10-fold cross-validation procedure.

Data used for the classifier	Classification performance (AUC)
{SNPs}	0.51
{Alc, Smk, Age, Pck}	0.60
{Fh}	0.66
{Fh, Alc, Smk, Age, Pck}	0.73
{SNPs}+{Alc, Smk, Age, Pck}	0.62
{SNPs}+{Fh}	0.64
{SNPs}+{Fh, Alc, Smk, Age, Pck}	0.73

The classification algorithm is Support Vector Machines (SVM). Only SNPs selected by Recursive Feature Elimination (RFE) are used. The following abbreviations are used for variable names: Age (age at interview), Smk (tobacco use), Alc (alcohol consumption), Fh (family history of esophageal cancer), and Pck (consumption of pickled vegetables). The “+” symbol in the Data column denotes that the analysis was performed by ensembling approach.

The experiments presented in this paper involved SVM classifiers. As we mentioned, the choice of classifier was based on empirical evidence suggesting that SVMs have superior performance in different high-dimensional “omics” datasets [19]–[21] as well as in SNP data [4] and they certainly outperform unsupervised classification methods such as PCA [27], [28]. However, one cannot preclude that there does not exist some classification methods that outperform SVMs in SNP array datasets. Future research will answer this question.

In conclusion, our findings suggest that several data analysis pitfalls of [8] led researchers to identify SNPs that are not statistically significant and to derive a severely biased estimate of classification performance of esophageal cancer patients and healthy controls on the basis of these SNPs. We also showed that environmental factors and especially family history of cancer (the latter may serve as proxy to both genetic and environmental factors) have a modest association with the disease. It is thus conceivable that other SNPs, not included in the assay employed, may be implicated in the disease. These results are consistent with the previous literature that emphasizes the importance of environmental factors on the causation of this complex disease [9], [10]. The results also underscore the importance of sound data analysis in genome-wide association studies.

## Supporting Information

File S1 Demonstration of Bias in Computation of P-Values
(0.08 MB DOC)

File S2 Integrated Analysis of Multiple Data Types
(0.09 MB DOC)
