# Supplementary material for: Effects of Environment, Genetics and Data Analysis Pitfalls in an Esophageal Cancer Genome-Wide Association Study
Source: PLoS One. 2007 Sep 26;2(9):e958. doi: 10.1371/journal.pone.0000958 (PMC1978529; doi:10.1371/journal.pone.0000958)
Supplement: File S1 — Demonstration of Bias in Computation of P-Values (0.08 MB DOC) [file pone.0000958.s001.doc]

***Supporting Information File S1:***

**Demonstration of Bias in Computation of P-Values**

We used a permutation method to analyze bias in computation of p-values in procedures GLM1 and GLM2. The underlying idea of this method is to create a null distribution where SNPs are known to be completely unrelated to the response variable and see how frequently procedures GLM1 and GLM2 find statistically significant associations [1,2].

First, we permute all subjects in the SNP data while leaving the response variable, family history of esophageal cancer, and alcohol consumption intact. Next, we apply a procedure for computation of p-values (GLM1 or GLM2) to the permuted SNP data and count how many SNPs are statistically significant at the 0.05 α-level with Bonferroni correction. The above two steps are repeated 1,000 times.

For all 1,000 permutations of SNP data, the procedure GLM1 found significant SNPs. The number of significant SNPs for each permutation was ranging from 185 to 1,938, on average 357. On the other hand, the procedure GLM2 found up to 3 significant SNPs in only 48 out of 1,000 permutations, that is the proportion of false positives expected at Bonferroni adjusted 0.05 α-level. These results clearly demonstrate that procedure GLM1 is biased while GLM2 is not.

**References**

1. Aliferis CF, Statnikov A, Tsamardinos I (2006) Challenges in the analysis of mass-throughput data: a technical commentary from the statistical machine learning perspective. Cancer Informatics 2: 133-162.

2. Good PI (2000) Permutation tests: a practical guide to resampling methods for testing hypotheses. New York: Springer.
