# Supplementary material for: Effects of Environment, Genetics and Data Analysis Pitfalls in an Esophageal Cancer Genome-Wide Association Study
Source: PLoS One. 2007 Sep 26;2(9):e958. doi: 10.1371/journal.pone.0000958 (PMC1978529; doi:10.1371/journal.pone.0000958)
Supplement: File S2 — Integrated Analysis of Multiple Data Types (0.09 MB DOC) [file pone.0000958.s002.doc]

***Supporting Information File S2:***

**Integrated Analysis of Multiple Data Types**

We used five methods for integrated analysis of multiple data types based on SVM classifiers.

- **Approach I**: We built two SVM classification models: one based on SNP data and another one based on environmental factors (and/or family history). The prediction for each subject is the average prediction of the two classification models.
- **Approach II**: Same as Approach I except that we used probabilistic SVM classifiers as described in [1].
- **Approach III:** We built two SVM classification models: one based on SNP data and another one based on environmental factors (and/or family history). The prediction for each subject is the average prediction of the two classification models *weighted by their performance on the training set*.
- **Approach IV**: Same as Approach III except that we used probabilistic SVM classifiers as described in [1].
- **Approach V:** We built a single classifier based on SNP data and environmental factors (and/or family history). This approach is commonly used in analysis of other types of high-throughput data, for example gene expression data [2].

The resulting classification performances of the five ensembling techniques are shown in the table below:

|  | **Classification performance (AUC)** | | | | |
| --- | --- | --- | --- | --- | --- |
| **Data used for the classifier** | *I* | *II* | *III* | *IV* | *V* |
| {SNPs} + {Alc, Smk, Age, Pck} | 0.62 | 0.55 | 0.61 | 0.54 | 0.53 |
| {SNPs} + {Fh} | 0.64 | 0.62 | 0.63 | 0.60 | 0.57 |
| {SNPs} + {Fh, Alc, Smk, Age, Pck} | 0.73 | 0.68 | 0.72 | 0.67 | 0.59 |

All methods confirm the conclusion that available SNP dataset does not provide an improvement of esophageal cancer classification performance compared to models based on 4 environmental variables and family history of the disease (see Table 1 in the manuscript).

**References**

1. Schölkopf B, Burges CJC, and Smola AJ (1999) Advances in kernel methods: support vector learning. Cambridge, Mass: MIT Press.

2. Li L, Chen L, Goldgof D, George F, Chen Z, et al. (2005) Integration of Clinical Information and Gene Expression Profiles for Prediction of Chemo-Response for Ovarian Cancer. Proceedings of the 27th Annual International Conference of the IEEE Engineering in Medicine and Biology Society, 2005 4818-4821.
